# Disseminated Nontuberculous Mycobacteria in HIV-Infected Patients, Oregon, USA, 2007–2012

**DOI:** 10.3201/eid2303.161708

**Published:** 2017-03

**Authors:** Cara D. Varley, Jennifer H. Ku, Emily Henkle, Sean D. Schafer, Kevin L. Winthrop

**Affiliations:** Tulane University, New Orleans, Louisiana, USA (C.D. Varley);; Oregon Health & Science University, Portland, Oregon, USA (C.D. Varley, J.H. Ku, E. Henkle, K.L. Winthrop);; Oregon Department of Human Services, Portland (C.D. Varley, E. Henkle, S.D. Schafer)

**Keywords:** mycobacterium avium, HIV/AIDS and other retroviruses, Oregon, United States, disseminated nontuberculous mycobacteria, bacteria, tuberculosis and other mycobacteria

## Abstract

We determined disseminated nontuberculous mycobacteria incidence in the HIV-infected population of Oregon, USA, during 2007–2012 by using statewide laboratory surveillance. We identified 37 disseminated nontuberculous mycobacteria cases among 7,349 patients with median annual incidence of 110/100,000 HIV person-years and the highest incidence in those with CD4 counts <50 cells/mm^3^ (5,300/100,000 person-years).

Nontuberculous mycobacteria (NTM) are ubiquitous in the environment and are increasingly implicated in human diseases worldwide. Disseminated NTM infections are seen exclusively among immunocompromised hosts, including those with AIDS (*1**–**3*). Historically, disseminated *Mycobacterium avium* complex (MAC) has been among the most common AIDS-presenting diagnoses, with a 49% case-fatality rate at 1 year (*2**,**4*). After 1987, the number of reported disseminated NTM infections grew with the prevalence of AIDS; in 1990, between 15% and 24% of US residents with AIDS had disseminated NTM (primarily MAC) (*4**–**6*).

After the US Public Health Service formally recommended prophylaxis for MAC prevention in 1993 and highly active antiretroviral therapy (HAART) became widespread in 1996, disseminated MAC in HIV patients declined. Incidence estimates of 10,000 cases/100,000 person-years in 1992 declined to 250 cases/100,000 person-years in 2007 in the United States; however, patient populations studied were limited to those established in specialty HIV care (*7**–**9*). Modern-day population-based estimates are lacking. Having access to statewide NTM isolates and HIV case reporting, we sought to generate a population-based estimate of disseminated NTM disease incidence among patients infected with HIV in Oregon, USA.

## The Study

We used statewide reported NTM isolate data from all laboratories in Oregon collected during 2007–2012. From these data, we defined disseminated NTM as a reported NTM isolate from blood, cerebrospinal fluid, bone marrow, lymph node, or other sterile site in an Oregon resident >18 years of age. In a sensitivity analysis, we expanded this definition to include isolates from nonsterile sites, specifically skin and urine, that could still represent disseminated disease. To identify cases of HIV-associated disseminated NTM, we linked patients with NTM isolates to the statewide HIV surveillance registry by name, date of birth, and sex using Registry Plus Link Plus (*10*). We reviewed all possible matches manually.

To calculate incidence, exposure time began with the date of HIV diagnosis. We censored cases at death, NTM diagnosis, or 1 year after the last reported CD4 or viral load result when the gap was >2 years between laboratory tests. Since 2006, Oregon law has required that laboratories report all CD4 counts and viral loads to the Oregon Health Authority. We calculated exposure time within CD4 ranges for each interval between reported CD4 counts until patients were censored. We calculated disseminated NTM incidence by CD4 count (closest to NTM diagnosis within preceding 3 months) and year with 95% CIs under the Poisson distribution in SAS version 9.4 (*11*). The Oregon Health Authority determined this project to be public health surveillance.

We identified 37 disseminated NTM cases among a population of 7,349 HIV-infected patients with 33,072 person-years of exposure; most were male (28, 75.7%), non-Hispanic (29, 78.4%), and white (26, 70.3%), with a median age of 40.0 years (range 22.7–59.4 years) (Table 1). Median time from HIV diagnosis to disseminated NTM was 2.1 years (range 0–20.7 years). Most cases were caused by MAC (35, 94.6%); 19 patients (52.8%, 1 outcome missing) died, with median survival of 0.3 years (range 0–5.8 years) after NTM diagnosis.

**Table 1:** Characteristics of 37 patients with HIV and disseminated NTM infection, Oregon, USA, 2007–2012*

Variable	Value
Race	
White	26 (70.3)
Black	6 (16.2)
Asian, Native American, Alaskan Native, Pacific Islander, **>**1 race	5 (13.5)
** Hispanic**	8 (21.6)
Female sex	9 (24.3)
Age at disseminated NTM diagnosis, y, median (range)	40 (22.7–59.4)
Deceased at time of data analysis	19 (52.8)
Time from HIV diagnosis to NTM diagnosis, y, median (range)	2.1 (0–20.7)
NTM species	
*M. aubagnense*	1 (2.7)
*M. avium*	35 (94.6)
*M. chelonae*	1 (2.7)
CD4 count, cells/mm^3^)†	10 (1–414)
<50	23 (74.2)
50–100	5 (16.1)
101–200	1 (3.2)
>200	2 (6.5)
Viral load, copies/mL, median (range)	131,446 (0–4,570,000)

*Values are no. (%) except as indicated. NTM, nontuberculous mycobacteria. †CD4 count missing for 6 patients.

Median CD4 count and viral load collected closest to NTM diagnosis were 10 cells/mm^3^ and 131,446 copies/mL, respectively. At the time of NTM isolation, 23 (74.2%) patients had CD4 counts <50 cells/mm^3^, 5 (16.1%) had CD4 counts 50−99 cells/mm^3^, 1 (3.2%) had a CD4 count 100−199 cells/mm^3^, and 2 (6.5%) had CD4 counts >200 cells/mm^3^. Of the 2 patients with CD4 counts >200 cells/mm^3^, both had previous CD4 counts >200 cells/mm^3^; 1 had an undetectable viral load, and the other had persistent viremia >200,000 copies/mL. The patient with a CD4 count of 100−199 cells/mm^3^ at the time of disseminated NTM diagnosis had a CD4 count of 32 cells/mm^3^ 9 months before diagnosis. 

CD4 testing frequency varied substantially in the 6,171 patients with reported CD4 counts during 2007–2012. Median time between CD4 count tests for those with testing intervals <2 years was 76 days (range 0–664 days). Six patients with disseminated NTM did not have CD4 counts during the 3-month period before diagnosis and were excluded from CD4-specific incidence.

The highest incidence of disseminated NTM (5,300/100,000 person-years) occurred in patients with CD4 counts <50 cells/mm^3^ (Table 2). Annual incidence ranged from 50/100,000 to 200/100,000 person-years (median 110/100,000), with no statistically significant difference over the 6 years.

**Table 2:** Incidence of disseminated nontuberculous mycobacterial disease, by CD4 count closest to diagnosis date, Oregon, USA, 2007–2012*

**Rate**	CD4 count, cells/mm^3^			
	<50, n = 677	50–100, n = 784	101–200, n = 1,697	>200, n = 5,827
**Incidence**/**100,000 person-years **(95% CI)	5,300 (3,360–7,950)	950 (310–2,210)	60 (0–310)	10 (0–30)

*n values indicate no. patients with >1 CD4 count in specified category.

An additional 10 cases of possible disseminated NTM infections were identified (4 skin, 1 wound, 1 urine, 2 abscess, 2 unknown site). Species included MAC (*7*), *Mycobacterium xenopi* (*1*), and unspeciated (*2*). All 10 cases occurred in white men of median age 45.2 years (range 32.0–62.8 years). None of these persons had a CD4 count <50 cells/mm^3^. The 9 cases with CD4 test results (1 did not have a CD4 test within 3 months of the study) were distributed evenly among the other 3 CD4 count groups (median 135 cells/mm^3^, range 92–352 cells/mm^3^). Two of these 10 patients had died by the time of the analysis.

## Conclusions

Limited studies have evaluated the incidence of disseminated NTM disease in persons with HIV in the HAART era (*7**,**9*). We evaluated the complete population of Oregon residents with HIV and NTM during 2007–2012 by merging 2 comprehensive statewide databases. Estimated annual incidence (0.11/100 person-years) was lower than estimates previously described in the HAART era (0.25–2/100 person-years) (*9**,**12**)*. During our study period, 32% (n = 1,996) of Oregon’s HIV-infected population had >1 CD4 count <200 cells/mm^3^ and 10.9% (n = 677) had >1 CD4 count <50 cells/mm^3^, indicating a relatively small population at theoretic risk for disseminated NTM. Of the 677 patients at highest risk, with >1 CD4 count <50 cells/mm^3^, disseminated NTM developed in only 3.4%.

Mortality rates from disseminated NTM remain high, with median survival and mortality rates similar to those shown in data reported previously (*4**,**6**,**7**,**12*). Three persons who had disseminated NTM had CD4 counts >99 cells/mm^3^. Most studies evaluating disseminated NTM are limited to patients with CD4 counts <99 cells/mm^3^; however, Nightingale et al. found that 7% of patients with a previous AIDS-defining illness and disseminated NTM disease had CD4 counts >99 cells/mm^3^ (maximum 441 cells/mm^3^) (*4*). Our findings are consistent with this and suggest that disseminated NTM should be considered in this population.

Oregon is a state with a relatively low HIV prevalence (143.1 cases/100,000 persons during 2007–2012), with a range of 71–79 HIV deaths annually and an average of 256 new cases yearly (*13*). Our data might have limited generalizability beyond Oregon, given that most patients living with HIV in Oregon have access to care; 87% of all patients in care have a suppressed viral load, and only 16% of AIDS Drug Assistance Program clients lack reported recent CD4 or viral load test results (*13*).

Analysis of incidence by CD4 count was also limited by variability in frequency of laboratory testing, which was determined by the treating clinicians. Six (16%) patients with disseminated NTM did not have CD4 counts taken 3 months before or 1 month after diagnosis. In addition, laboratory results could have been missing in Oregon residents who receive HIV care outside the state.

Our clinical data were limited to laboratory results; therefore, we were unable to analyze the impact of antiretroviral therapy, antimicrobial prophylaxis or treatment, and access to care. Given the rarity of this infection in our cohort, we suspect that prophylaxis is routinely being used in those with CD4 count <50 cells/mm^3^; however, mortality rates continue to be high in those in whom disseminated NTM infections develop.
